# Asymptomatic migration of ventral mesh for incisional hernia into the small intestine: A case report

**DOI:** 10.1002/ccr3.2212

**Published:** 2019-05-29

**Authors:** Evangelia Triantafyllou, Iraklis Anastasiadis, Dimitrios Konstantinidis, Athanasios Syllaios, Efrosyni Gerovasileiou, Orestis Tsiripidis

**Affiliations:** ^1^ Surgical Department Mpodosakeio General Hospital of Ptolemaida Ptolemaida Greece; ^2^ First Department of Surgery Laikon Hospital Athens Greece; ^3^ University General Hospital of Larissa Larissa Greece

**Keywords:** abdominal, asymptomatic, hernia, mesh, migration, surgery

## Abstract

Even though mesh migration is a rare complication, it must be considered in the differential diagnosis when investigating abdominal pain and digestive complications in patients with history of abdominal operations.

## 1 INTRODUCTION

Incisional hernia is considered to be one of the most common complications of abdominal surgery, with an incidence rate of 5.2% at 12 months and 10.3% at 24 months.^1^ Nowadays, synthetic mesh is commonly used in order to repair the abdominal wall and eliminate tension thus prevent any recurrences. Complications have been reported over the years due to the use of meshes. In this case report, we present the case of an asymptomatic intraluminal migration of a synthetic mesh used for the repair of a postoperative ventral hernia repair.

## 2 CASE REPORT

**Figure 1:**
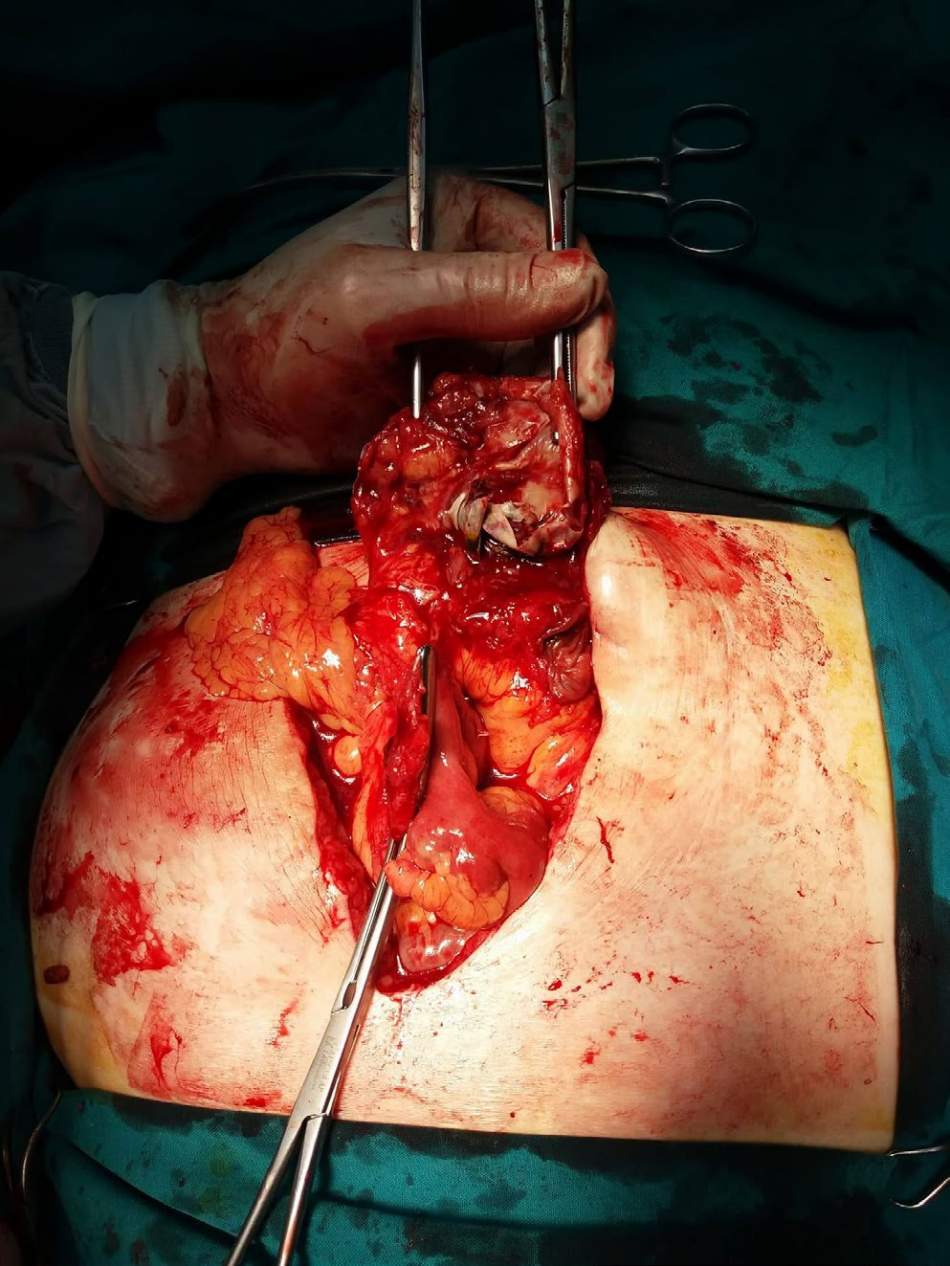
Intraoperative findings

A 72‐year‐old woman was referred to our outpatient clinic because of a recurrent incisional hernia. Patient history includes a well‐controlled hypertension (treated for 7 years). Surgical history includes an umbilical hernia, operated 5 years ago, as well as a recurrence of the umbilical hernia, treated as an incisional hernia, 2 years ago. Both operations were performed at another surgical department; as a result, information concerning the techniques used for the repairs was unavailable. The patient was scheduled for a recurrent hernia repair. Prior to surgery, she was otherwise asymptomatic, with no signs of bowel obstruction. Physical examination revealed a periumbilical defect of about 7 cm in the abdominal wall. Intraoperatively, an enteroenteric fistula in close relation to the abdominal wall was discovered, as well as a palpable hard mass which was located next to the fistula. Adhesions were also present. During adhesiolysis, the small intestine was incised and a mesh was discovered inside the lumen (Figure 1). Partial enterectomy (about 20 cm long) was performed, and the continuity was restored with an end‐to‐end anastomosis by hand. The recurrent ventral hernia was restored with simple suturing and closing of the defect, since the risk of foreign material infection was augmented, because of the small bowel manipulations. Postoperatively, oral fluid intake was encouraged on the first day and patient was discharged from our clinic on the sixth postoperative day.

## 3 DISCUSSION

An incisional hernia is a common complication of abdominal surgery. The use of meshes is now the treatment of choice. A mesh facilitates the application of tension‐free techniques. Nonetheless, complications related to the artificial materials used have been reported. These include seroma, foreign body reaction, organ injury, infection, mesh rejection, fistulas, and mesh migration.^2^

Even though mesh migration is a rare complication, it must be considered in the differential diagnosis when investigating abdominal pain and digestive complications in patients with history of abdominal operations. Migration of mesh into the urinary bladder might present with hematuria^3^ or recurrent urinary tract infections.^4^ Ghandhi et al^5^ mention two mechanisms to explain the migration of mesh “Mechanical migration primarily occurs when an inadequately secured mesh traverses along adjoining paths of least resistance or when a relatively secure mesh is displaced by external forces. Secondary migration, on the other hand, occurs through trans‐anatomical planes and is the result of erosions triggered by foreign body reaction.” It is also noted that the presence of inflammatory granulation tissue at the site of the migration as described by histopathological reports supports this mechanism. Dieter reported in 1999 a case of mesh migration into the scrotum.^6^ In addition, a case of spontaneous evacuation per rectum^7^ has been reported, as well as ventral hernia mesh migration with splenosis mimicking a gastric mass.^8^

In this case report, we describe a case of an asymptomatic ventral mesh migration which resulted in the erosion of the intestinal wall and the formation of an enteroenteric fistula. To our knowledge, this is the first case, an asymptomatic migration of mesh discovered as an incidental finding in a programmed hernia recurrence repair.

As it has been already described by Aziz et al,^2^ the biomaterial used is an important factor that affects the extent and degree of mesh integration with the surrounding tissue.^9^ The positioning technique and tissue degeneration are additional important factors to be taken into consideration.^10^, ^11^ As a consequence, the more the mesh is integrating in its surroundings, the higher the probability of hollow viscus erosion was. It is also stated that low‐weight mesh shows significantly less cellular proliferation and foreign body reaction than traditional prolene mesh. As far as migration is concerned, it is believed that the fixation method also affects migration rates by altering the degree of movement and tensile strength of the mesh. Other important factors influencing the migration rate is the size, shape, and positioning.^9^ Direct contact with the abdominal organs should be avoided, either by positioning the mesh in the preperitoneal space or by fixating the omentum to secure maximum organ coverage. New techniques with dynamic implants and fixation‐free prosthesis have also been developed and are currently tested. The use of tentacle‐shaped implant ensures mesh stability and broad defect overlap, with a very low complication rate.^12^

## CONFLICT OF INTEREST

None declared.

## AUTHOR CONTRIBUTIONS

IA: received the patient in our outpatient department. OT: was the principal surgeon. ET: drafted the manuscript and was also a major contributor in revising the manuscript. DK: revised the manuscript critically for important intellectual content. ET and IA: analyzed and interpreted the patient data. AS: assisted in drafting the manuscript. ET: served as an auxiliary surgeon and had significant contribution to conception and design of the manuscript. OT: was responsible for the overall treatment of the patient, revised critically the manuscript, and has given final approval of the version to be published. All authors read and approved the final manuscript.
